# Pharmacologic inhibition of IL11/STAT3 signaling increases MHC-I expression and T cell infiltration

**DOI:** 10.1186/s12967-023-04079-6

**Published:** 2023-06-26

**Authors:** Wenjun Xiong, Yuehong Chen, Chaoting Zhang, Jin Li, Haipeng Huang, Yu Zhu, Guangxu Deng, Junhong Cheng, Yixiong Lin, Zhimin Shi, Tingyu Mou

**Affiliations:** 1 Department of Gastrointestinal Surgery, Guangdong Provincial Hospital of Chinese Medicine, The Second Affiliated Hospital of Guangzhou University of Chinese Medicine, Guangzhou, China; 2 Department of General Surgery, Nanfang Hospital, Southern Medical University, No. 1838 Guangzhou Avenue North, Guangzhou, Guangdong China; 3 Guangdong Provincial Key Laboratory of Precision Medicine for Gastrointestinal Tumor, Guangzhou, China

**Keywords:** Colorectal cancer, IL11/STAT3 signaling, CD8^+^ T infiltration, IFNγ/STAT1, MHC-I molecules

## Abstract

**Background:**

Recent studies have discovered an emerging role of IL11 in various colitis-associated cancers, suggesting that IL11 mainly promotes tumor cell survival and proliferation in regulating tumorigenesis. Herein we aimed to reveal a novel function of IL-11 through STAT3 signaling in regulating tumor immune evasion.

**Methods:**

AOM/DSS model in *Il11*^−/−^ and *Apc*^min/+^/*Il11*^−/−^ mice were used to detect tumor growth and CD8^+^ T infiltration. STAT1/3 phosphorylation and MHC-I, CXCL9, H2-K1 and H2-D1 expression were detected in MC38 cells and intestine organoids treated with/without recombinant IL11 to explore effect of IL11/STAT3 signaling, with IL11 mutein used to competitively inhibit IL11 and rescue inhibited STAT1 activation. Correlation between IL11 and CD8^+^ T infiltration was analyzed using TIMER2.0 website. IL11 expression and survival prognosis was analyzed in clinical data of patient cohort from Nanfang Hospital.

**Results:**

IL11 is highly expressed in CRC and indicates unfavorable prognosis. IL11 knockout increased CD8^+^ T cell infiltration and reduced intestinal and colon formation. Tumors were significantly suppressed while MHC-I and CXCL9 expression for CD8^+^ T infiltration were remarkably increased in the tumor tissues of *Apc*^min/+^/*Il11*^−/−^ mice or *Il11*^−/−^ mice induced by AOM/DSS. IL11/STAT3 signaling downregulated MHC-I and CXCL9 by inhibiting IFNγ-induced STAT1 phosphorylation. IL11 mutein competitively inhibit IL11 to upregulate CXCL9 and MHC-I in tumor and attenuated tumor growth.

**Conclusions:**

This study ascribes for a new immunomodulatory role for IL11 during tumor development that is amenable to anti-cytokine based therapy of colon cancer.

**Supplementary Information:**

The online version contains supplementary material available at 10.1186/s12967-023-04079-6.

## Background

Colon cancer remains to be one of the leading causes of cancer-related death globally [1], and inflammatory bowel disease (IBD) is one of the critical factors in CRC carcinogenesis [2]. In patients with IBD, such as ulcerative colitis (UC), the risk of CRC development is approximately three- to fivefold increased to that of the general population [3]. Long-standing UC predisposes to development of colitis-associated cancer (CAC) [4]. It has been proposed that inflammatory factor released during chronic inflammation, including a variety of cytokines and chemokines, propagate a localized inflammatory response and also promote cell proliferation or survival, thereby promoting oncogenesis [5]. Studies utilizing knockout mice have begun to unravel the complex interplay between the neoplastic and stromal cells and have highlighted pivotal roles of inflammatory cytokines [6].

IL11 is a member of the IL-6 family of cytokines that utilizes the GP130 signaling pathway shared by other cytokines in the same family [7]. Various cell types, including fibroblasts [8], osteoblasts, neurons, and endothelial cells, produce IL11 in vivo [9]. IL11 is best known for its role in hematopoiesis since it promotes megakaryopoiesis, erythropoiesis and thrombopoiesis [10]. In view of these characteristics, recombinant human IL11 protein has been applied in clinical treatment of chemotherapy-induced thrombocytopenia [11]. Traditionally regarded as an anti-inflammatory cytokine, IL11 also demonstrates its proinflammatory role. In the intestine, IL11 can stimulate the proliferation of epithelial cells and reduce the apoptosis of mature epithelial cells and their progenitor cells [12]. On the contrary, hepatocytes undergo apoptosis under the action of IL11 [13].

Although IL11 was initially found to be a hematopoietic and inflammatory cytokine, the use of IL11 receptor alpha null mice (*Il11ra1*^KO^) has revealed a more prominent role of IL11 compared to IL-6 during the progression of sporadic and inflammation-associated colon and gastric cancers [14]. IL11 correlates with progression of various cancers, including colorectal cancer [8, 15], breast cancer [16, 17], hepatocellular carcinoma [18], gastric carcinoma [19] and renal cell carcinoma [20]. Moreover, IL11 also implicates poor survival in non-small cell lung adenocarcinoma [21]. Although it has been revealed that IL11 was closely related to the initiation and progression of cancer, there are few studies specify its regulatory mechanism.

IL11 binds to its specific transmembrane receptor, IL11 receptor alpha (IL11Ra), and the IL11/IL11Ra dimeric complex potentially interacts with GP130 to form a tetrameric complex [7]. The formation of this complex initiates signaling through intracellular Janus tyrosine kinases (JAK) family by activating signaling molecules, signal-transducer and activated of transcription-3 (STAT3) [22]. Besides activation of STAT3, engagement of GP130 also triggers signaling through the MAPK pathway and PI3K–AKT–mTORC1 pathway [23, 24]. By activating these pathways, previous studies revealed tumor-cell intrinsic activities of IL11 in cell proliferation, survival, motility, invasion and metastasis. More recently, an immune-modulatory role of IL11 has been reported through its suppressive effect on host CD4^+^ T cells in the tumor microenvironment [25].

We noticed that most of the previous studies mainly focused on IL11 effect on cell proliferation, invasion and metastasis. Meanwhile, the experiment was mainly carried out in Il11ra-null mice. In the present study we describe a hitherto unrecognized immune suppressing role of IL11 in initiation and progression of colorectal cancer based on IL11 knockout mice. We revealed an unprecedented role of IL11 in regulating the expression of MHC-I which was critical for the recognition of cytotoxic T lymphocyte (CTL). Furthermore, we tested the therapeutic effect of mIL11 mutein, a potent IL11 signaling antagonist that has a 20-fold higher affinity than IL11 for binding to IL11Ra [26].

## Methods

### Clinical specimens

Fresh surgical specimens of CRC tissues and matched adjacent normal mucosa of 101 patients were collected from Nanfang hospital and stored in liquid nitrogen until further analysis. All tissues were histologically confirmed as colon adenocarcinoma. None of these patients received chemotherapy or radiotherapy before operation. All tissues and classifications were performed based on the system of the International Union Against Cancer. The study protocols concerning human subjects are consistent with the principles of the Declaration of Helsinki and approved by the Ethics Committee of Nanfang Hospital. All patients given informed consents.

### Cell lines and mice

Mice CRC cell lines MC38 and CT26, human CRC cell lines HT29 and RKO were obtained from ATCC (Manassas, VA, USA). Cells were cultured in Dulbecco’s modified Eagle’s medium (DMEM) containing 10% heat-inactivated fetal bovine serum (FBS). The cells were maintained at 37 °C in a humidified incubator containing 5% CO_2_. Mycoplasma contamination was detected every month via PCR.

*Il11*^−/−^ mice was established by CRISPR/Cas9-derived knockout (KO) 1st and 5th exon of *Il11* gene and confirmed using PCR genotyping. Wild-type (WT) C57BL/6 mice were purchased from the Central Laboratory of Animal Science of Southern Medical University. *Apc*^min/+^mice were purchased from Jackson’s laboratory. *Stat3*^*fl/fl*^ mice were kindly gifted by Prof. Guoxin Li, Southern Medicine University. Mice were raised in a specific pathogen-free environment under suitable temperature and light-controlled room with free access to food and water. All studies were performed in male mice unless otherwise indicated. Animal related research protocols are consistent with the U.S. Public Health Service Policy on Use of Laboratory Animals, and were approved by the Ethics Committee on Use and Care of Animals of Southern Medical University.

### Colitis-associated colorectal cancer mice model

C57BL/6 or *Il11*^−/−^ mice (*n* = 8 per group) were intraperitoneally (i.p.) injected 10 mg/kg body weight (B.W.) of AOM, and kept on regular diet and water for 7 days. After 7 days, mice received water with 2.5% DSS for 7 days, and resume to regular diet and water. Afterwards, mice received a second 7-day administration of DSS on day 28, and on day 49 a third. All mice were sacrificed and analyzed on day 86.

### Mice colonoscopy

At indicated time points, mice were anesthetized by inhaling 1.5 to 2% isoflurane (RWD Life Science Co., Ltd, Shenzhen, China). Optical colonoscopy was performed using Karl Storz Image HD Camera System (Tuttlingen, Germany). Total intestines and colons were dissected, stained with methylene blue. Tumors on intestines and colons were counted, measured and photographed, then fixed in formalin and embedded in paraffin for further immnuohistological assessment.

### Subcutaneous tumor model, CD8α antibody depletion and IL11 recombinant/IL11 mutein administration

2 × 10^6^ per mice of MC38 or CT26 cells were subcutaneously transplanted into right back flank of C57BL/6 or Balb/c mice respectively (*n* = 6/group). Tumor length and width were measured and calculated (tumor volume = length × width^2^ × 0.5). Mice were sacrificed when tumors reached 2000 mm^3^ in volume or when sign of ulceration was evident. Tumors were dissected, measured and photographed at indicated time, then embedded in OCT or fixed with formalin and embedded in paraffin for further assessment. For CD8^+^ T cells depletion, 200 μg/dose anti-CD8α (BE0061, BioXcell) was injected i.p. twice weekly before tumor cell injection. Where indicated, IL11 recombinant was i.p. injected 10 mg/kg B.W. every 2 days after tumor cell injection, and IL11 mutein was i.p. injected 10 mg/kg B.W. every 2 days after tumor cell injection.

### MC38 intraperitoneal injection model and IL11 mutein administration

For survival analysis, 2 × 10^6^ per mice of MC38 cells were injected i.p. into C57BL/6 mice (*n* = 10 per group). Mice were sacrificed when weight loss more than 20%. For IL11 mutein administration, 10 mg/kg B.W. of IL11 mutein was injected i.p. every 2 days after tumor cell injection.

### Immunohistochemistry (IHC) and immunofluorescence (IF) staining

For IHC staining, tissue slides were deparaffinized and hydrated, incubated with antibody anti-Ki-67 (1:500, #550609, BD Science), overnight at 4 °C. For negative controls, the antibodies were replaced with normal non-immune serum. For cell IF staining, a multiplexed tyramide signal amplification method (TSA; PerkinElmer, Inc., US) was performed on 5 μm sections of frozen tissues to detect CD8 positive cell. Staining was performed using anti-CD8 (1:200, #100716, Biolegend). Secondary antibodies and fluorescent reagents used for CD8 detection were goat anti-rat IgG with fluorescein-Cy3 (Perkin Elmer). DAPI was used to stain nucleus. Cover slips were mounted using Prolong Gold anti-fade medium. Slides were imaged using Carl Zeiss LSM880 Inverted laser scanning confocal microscope. The total number of CD8-positive cells per 5 microscopic fields (400×) was calculated.

### Immunoblot analysis

Cultured cells or organoids were lysed with lysis buffer and quantified with BCA Protein Assay Kit (Thermofisher, 23225). Equal amount of protein extracts was separated by electrophoresis and then transferred to PVDF membrane (IPFL00010, Merck Millipore). After 5% fully skimmed milk blocking, the PVDF membrane were incubated with the primary antibody anti-GAPDH (1:1000, 60004-1-Ig, Proteintech), anti-p-STAT3 (1:100, 8478S, Cell Signaling Technology), anti-STAT3 (1:100, 8478S, Cell Signaling Technology), anti-p-STAT1 (1:100, 8478S, Cell Signaling Technology), and anti-STAT1 (1:100, 8478S, Cell Signaling Technology). Signals were detected using horseradish peroxidase (HRP)-conjugated secondary antibodies and Super Signal West Femto Chemiluminescent Substrate (34096, Thermo Fisher Scientific). Images were captured using the Image Lab Software (Tanon 5200, ver 1.00). Protein expression level was semi-quantified by Tanon Image analyze system (version 1.0) and normalized to corresponding GAPDH.

### Total RNA extraction and real-time quantitative PCR

Cell lines, mice tumor tissues, fresh CRC tissues and matched adjacent normal mucosa were isolated using Trizol reagent (TaKaRa, Dalian China) following manufacturer's instruction. cDNA synthesis was performed according to the instruction of PrimeScript™ RT reagent Kit (TaKaRa, Dalian China). qRT-PCR was carried out using SYBR Premix Ex Taq™ II (TaKaRa, Dalian China) and 7500-fast instrument (Applied BioSystems). Data were normalized to the mean Ct values of housekeeping gene GAPDH and presented as 2^−ΔΔCt^. Primers used for qRT-PCR were supplemented in Additional file 4: Table S2.

### Crypt isolation, organoid culture and transfection

Intestinal crypts were isolated from C57BL/6 or *Stat3*^*fl/fl*^ mice, based on previously published protocols with modification [27]. Briefly, small intestines from mice were dissected longitudinally, and washed with cold phosphate-buffered saline (PBS). The intestine was cut into 5 mm pieces and washed in a 50 mL conical centrifuge tube containing 30 mL washing buffer (1 U/ml of penicillin, 1 μg/ml of streptomycin, and 2.5 ng/ml of amphotericin B in PBS) for two times. Segments were transferred into 30 ml EDTA chelation buffer (15 mM EDTA in washing buffer), shaken on a tube rotator for 30 min, and then oscillated vigorously for 2 min on VORTEX-5 (Kylin-Bell). Dissociated intestinal crypts were filtered through 70 μm strainers. Collected supernatant were rotated at 200 × *g* for 10 min at 4 °C to collect intestinal crypts. Crypt pellets were resuspended with organoid growth medium, counted, and embedded in Matrigel (growth factor reduced, phenol red free; BD Biosciences) on ice.

Crypts were embedded into 50 μl/well Matrigel (BD Biosciences) at in 24-well plates, and were overlaid with 500 μl ENR culture medium, containing 2 mM Glutamax, 10 mM HEPES, 100 U/ml penicillin, 100 μg/ml streptomycin (Invitrogen), 1 mM *N*-acetyl cysteine (Sigma), B27 supplement (Invitrogen), N2 supplement (Invitrogen), 50 ng/ml mouse EGF (Peprotech), 100 ng/ml mouse Noggin (Peprotech) and 100 ng/ml R-spondin-1 (R&D Systems) or 10% human R-spondin-1-conditioned medium from R-spondin-1-transfected HEK 293T cells.

Organoid transfection was conducted as described protocol [28]. Briefly, organoids were cultured in WENR medium (Wnt3a and CHIR99021 were added in ENR medium) to enrich for stem cells before transfection. Organoids were trypsinized for 10 min at 37 °C to obtain single cell suspension. Adenovirus together with polybrene were mixed in 1 ml WENR medium and added into 24-well plates. After incubated for 6 h, cells were resuspended in 25% WENR medium-containing Matrigel. After 48 h, puromycin or neomycin was added to each well to screen transfected cells. Adenovirus encoding Cre were purchased from WZ bioscience (Shandong, China).

### Flow cytometry

Indicated MC38 cells were treated to IL11 (100 ng/ml) or IFNγ (50 ng/ml) for 24 h. Washed cells were resuspended in staining buffer (PBS with 1% BSA). Cells were incubated with Fc Blocker (clone 93; Biolegend) for 20 min on ice. Subsequently, anti-H-2Kd/2Dd Class I (1:200, #12-5998-81, ebioscience) antibody were added and staining was continued for 40 min on ice. After a washing step, cytometry sample acquisition was performed on a LSRFortessa X-20 (BD), and analysis was performed using FlowJo software (TreeStar).

### Statistics

Statistical parameters are all shown in figure legends. Statistical analysis was performed using nonparametric two-tailed *t* test or two-way ANOVA in GraphPad Prism. The survival data were analyzed by using Log-rank (Mantel-Cox) test. Correlation analysis between IL11 and CD8^+^ T infiltration was performed using TIMER2.0 website (http://timer.cistrome.org/) [29–31]. Unless otherwise described, error bars stand for standard error of the mean. *P < 0.05; **P < 0.01; ***P < 0.001; ****P < 0.0001.

## Results

### IL11 knockout increases CD8^+^ T cell infiltration and reduces intestinal and colon tumorigenesis

**Fig. 1:**
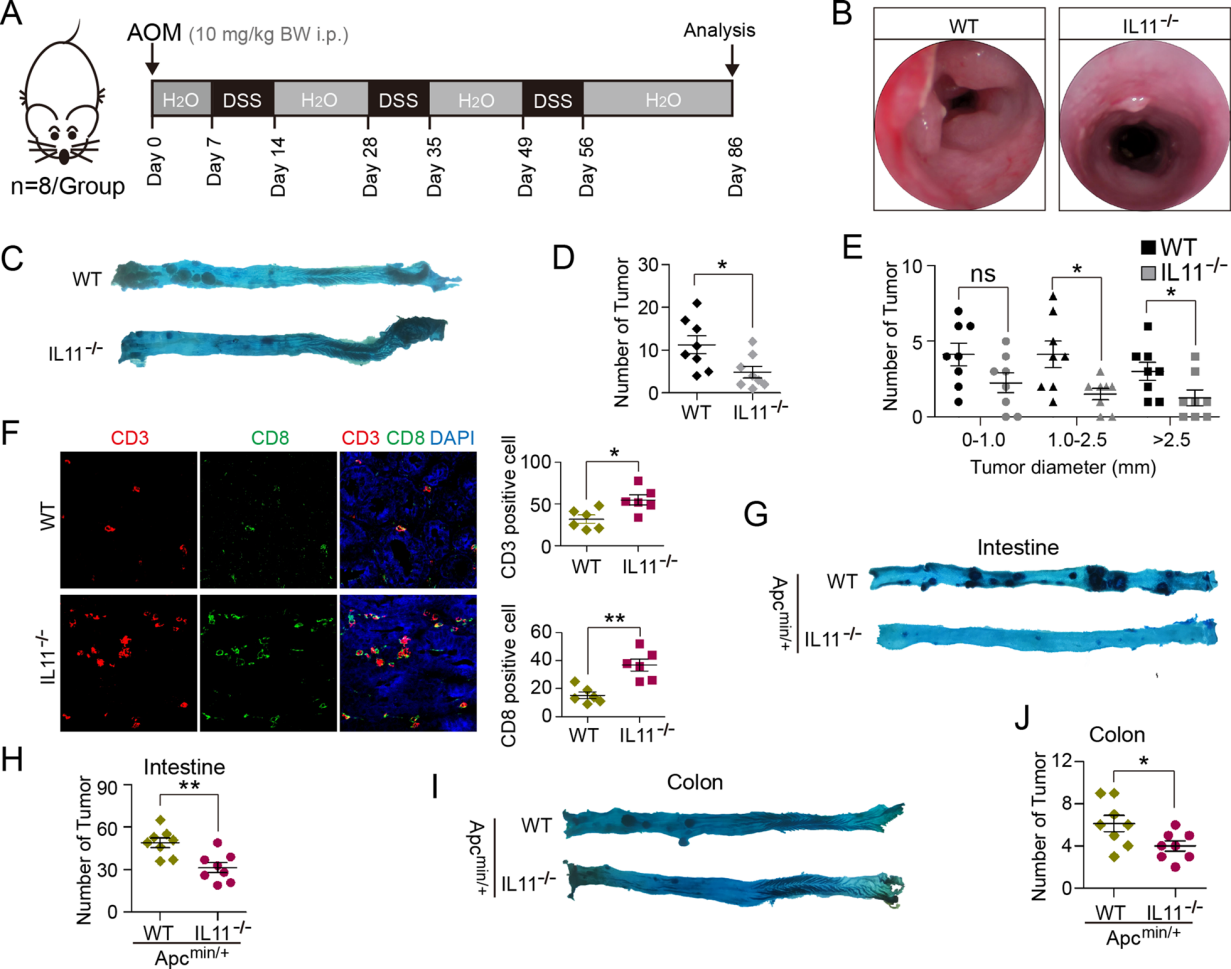
IL11 knockout increases CD8^+^ T cell infiltration and reduces intestinal and colon tumorigenesis. **A** Schematics of AOM/DSS administration and analysis time points (*n* = 8 mice/group). C57BL/6 or *Il11*^−/−^ mice (*n* = 8 per group) were intraperitoneally (i.p.) injected with 10 mg/kg body weight (BW) of AOM, and kept on regular diet and water for 7 days. After 7 days, mice received water with 2.5% DSS for 7 days, and resume to regular diet and water. Afterwards, mice received a second 7-day administration of DSS on day 28, and on day 49 a third. All mice were sacrificed and analyzed on day 86. **B** Colonoscopy images of AOM/DSS-induced CAC in WT and *Il11*^−/−^ mice on day 86. **C** Representative images of WT and *Il11*^−/−^ colon and rectum autopsy on day 86 (stained with methylene blue). **D** Tumor count of WT and *Il11*^−/−^ colon and rectum (*n* = 8 mice/group). *P < 0.05, *t* test. **E** Tumor diameter (mm) of WT and *Il11*^−/−^ colon and rectum (*n* = 8 mice/group). ns, no significance; *P < 0.05, *t* test. **F** Immunofluorescence and cell counts of CD3 (red), CD8 (green) and DAPI for nuclei (blue) in WT and *Il11*^−/−^ colon tumors (*n* = 6 mice/group). Scale bar, 50 μm. *P < 0.05, **P < 0.01, *t* test. **G** Representative images of 14-week old *Apc*^min/+^ and *Il11*^−/−^/*Apc*^min/+^ intestine autopsy (stained with methylene blue). **H** Tumor count of *Apc*^min/+^ and *Il11*^−/−^/*Apc*^min/+^ intestine (*n* = 8 mice/group). **P < 0.01, *t* test. **I** Representative images of 14-week old *Apc*^min/+^ and *Il11*^−/−^/*Apc*^min/+^ colon and rectum autopsy (stained with methylene blue). **J** Tumor count of *Apc*^min/+^ and *Il11*^−/−^/*Apc*^min/+^ colon and rectum (*n* = 8 mice/group). *P < 0.05, *t* test

*Il11*^−/−^ mice was established by CRISPR/Cas9-derived knockout (KO) 1st and 5th exon of *Il11* gene and confirmed using PCR genotyping (Additional file 1: Fig. S1A, B). AOM/DSS was administrated to induced colitis-associated colorectal cancer (CAC) in *Il11*^−/−^ and wild-type (WT) mice (Fig. 1A). Under endoscopy (Fig. 1B) and autopsy inspection (Fig. 1C) at indicated times, *Il11*^−/−^ mice formed less and smaller tumors after induction (Fig. 1D, E). Immune fluorescence (IF) analysis showed that tumor infiltrating CD3^+^ and CD8^+^ cells significantly increased in *Il11*^−/−^ tumors (Fig. 1F). We then crossbred *Apc*^min/+^ mice, a classical spontaneous tumor model, with *Il11*^−/−^ mice, to generate *Il11-*deficient environment in *Apc*^min/+^ mice. Compared with *Apc*^min/+^, *Il11*^−/−^/*Apc*^min/+^ mice formed less tumors in both intestine (Fig. 1G, H) and colon at 14-week old (Fig. 1I, J).

**Fig. 2:**
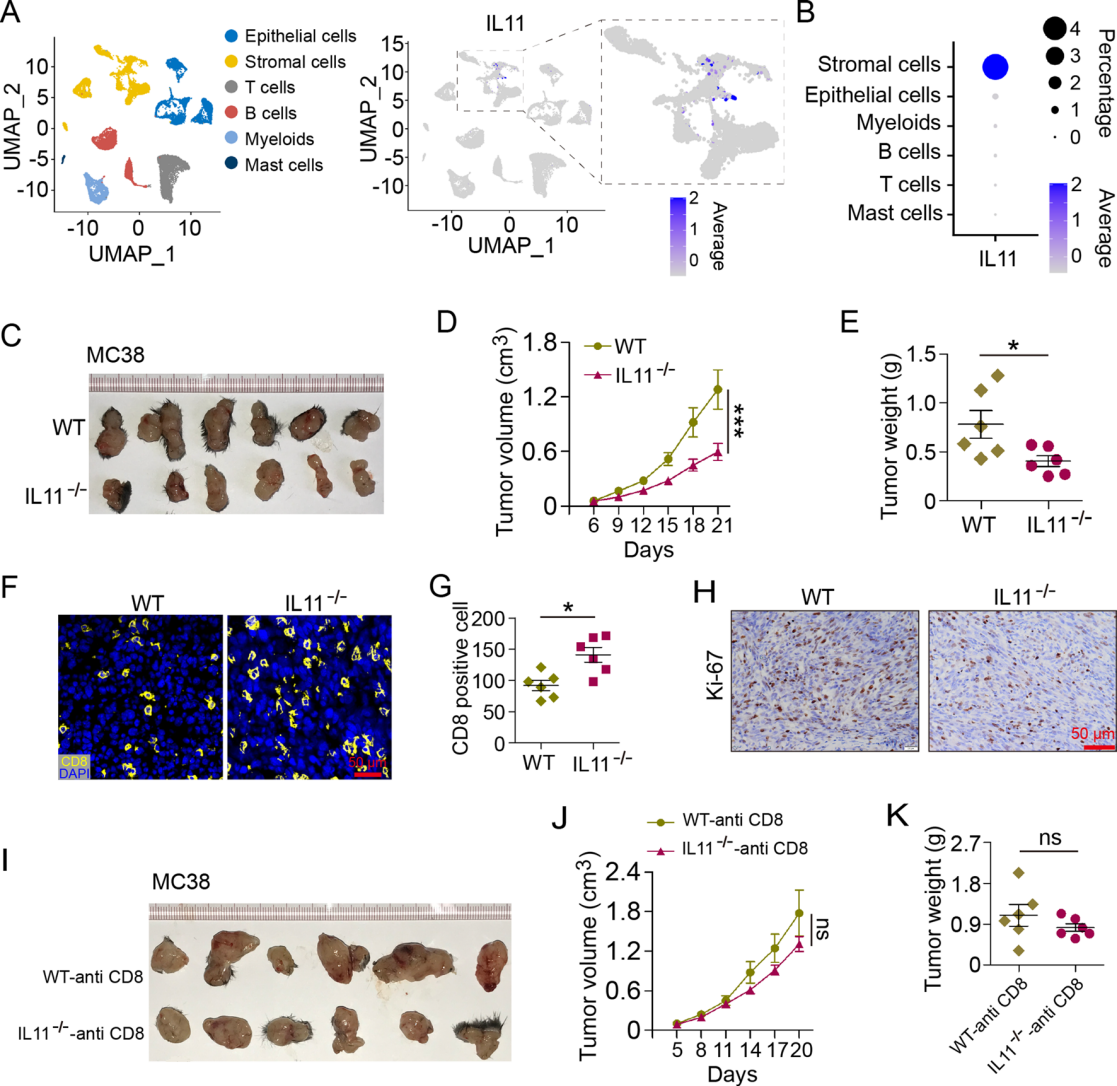
IL11 exerts pro-tumor effect through decreasing CD8^+^ T infiltration. **A** Uniform Manifold Approximation and Projection (UMAP) of single-cell sequencing data GSE144735. Cells were grouped and colored in 6 clusters using Seurat. Expression and distribution of IL11^+^ cells were highlighted. **B** Percentage and average expression of IL11 in 6 cell types in GSE144735. **C** Representative images of MC38 subcutaneous tumors in WT and *Il11*^−/−^ mice (*n* = 6 mice/group). **D** Tumor volume (cm^3^) of MC38 subcutaneous tumors in WT and *Il11*^−/−^ mice (*n* = 6 mice/group). ***P < 0.001, two-way ANOVA. **E** Tumor weight of MC38 subcutaneous tumors in WT and *Il11*^−/−^ mice (*n* = 6 mice/group). *P < 0.05, *t* test. **F**, **G** Immunofluorescence and cell counts of CD8 (yellow) and DAPI for nuclei (blue) of MC38 subcutaneous tumors in WT and *Il11*^−/−^ mice (*n* = 6 mice/group). Scale bar, 50 μm. *P < 0.05, *t* test. **H** Ki-67 staining of MC38 subcutaneous tumors in WT and *Il11*^−/−^ mice. Scale bar, 50 μm. **I** Representative images of MC38 subcutaneous tumors in CD8α-depleted WT and *Il11*^−/−^ mice (*n* = 6 mice/group). **J** Tumor volume (cm^3^) of MC38 subcutaneous tumors in CD8α-depleted WT and *Il11*^−/−^ mice (*n* = 6 mice/group). ns, no significance, two-way ANOVA. **K** Tumor weight of MC38 subcutaneous tumors in CD8α-depleted WT and *Il11*^−/−^ mice (*n* = 6 mice/group). ns, no significance, *t* test

In light of previous reports [9] and analysis of CRC single-cell sequencing data (GSE144735; Fig. 2A, B) [32], we learned that IL11 is predominantly expressed by stromal cells other than tumor cells. MC38 cells were subcutaneously inoculated into *Il11*^−/−^ mice, which presented a slower growing curve and smaller tumor weight compared with those in WT mice (Fig. 2C–E). IF analysis of subcutaneous tumors showed increased CD8^+^ cell infiltration in *Il11-*deficient tumor environment (Fig. 2F, G). Immunohistochemistry staining showed less Ki-67^+^ proliferating cells in *Il11-*deficient tumor environment (Fig. 2H). To confirm CD8^+^ T cells’ role in such context, *Il11*^−/−^ mice were subjected to CD8α depletion before MC38 subcutaneous injection, of which tumor growth and weight showed no statistical difference compared with WT counterparts (Fig. 2I–K). These results indicated pro-tumor effect of IL11 through decreasing CD8^+^ T infiltration.

### IL11 activates STAT3 to inhibit IFNγ-induced STAT1 signaling and MHC-I expression

**Fig. 3:**
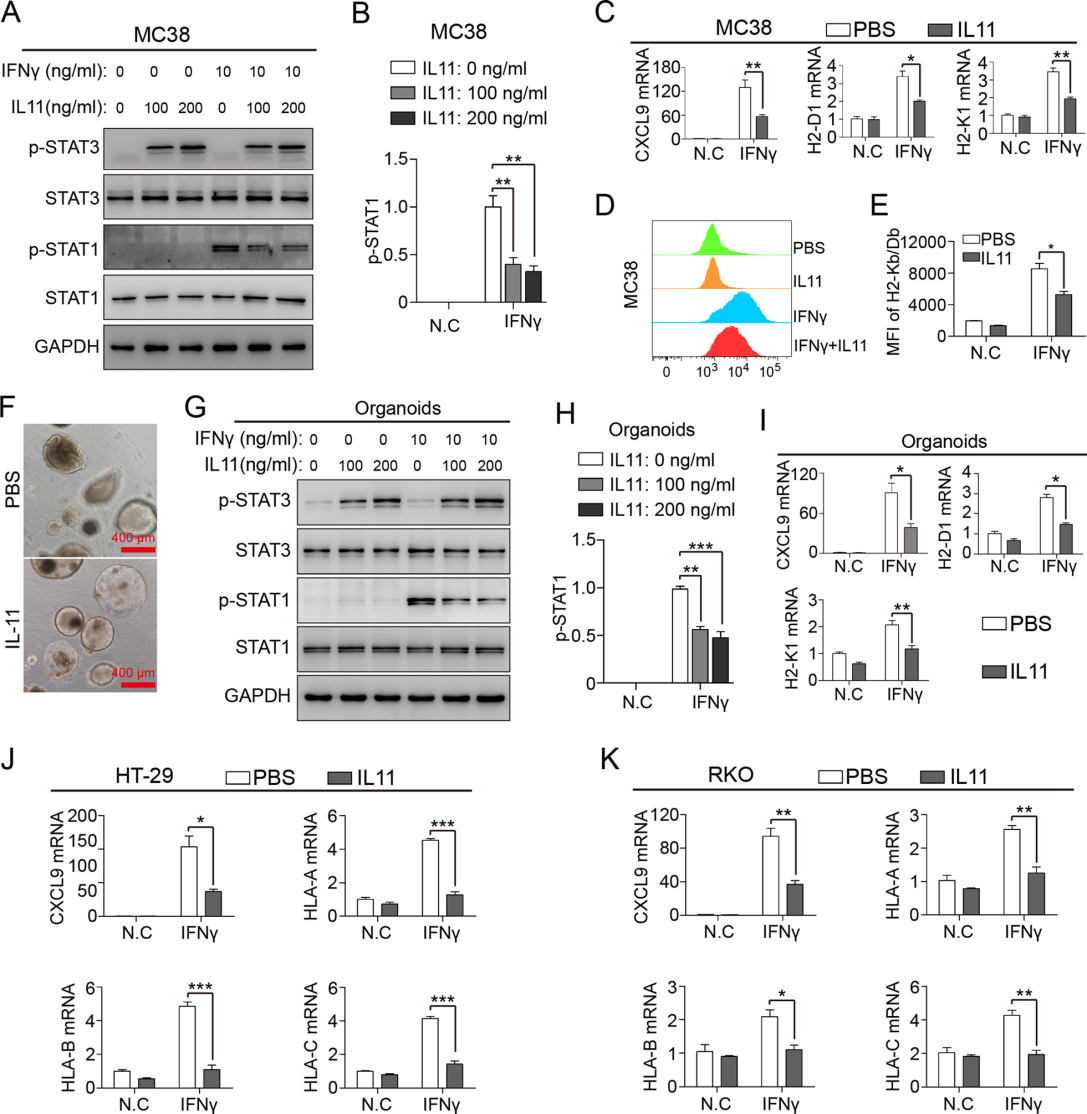
IL11 activates STAT3 to inhibit IFNγ-induced STAT1 signaling and MHC-I expression. **A**, **B** Immunoblot of STAT1/3 (total and phosphorylated) in IL11-treated (100/200 ng/ml) MC38 with/without IFNγ induction (10 ng/ml). GAPDH as internal reference. STAT1 phosphorylation ratio was calculated. Data presented as means ± SD from three independent experiments. *P < 0.05, **P < 0.01, ***P < 0.001, *t* test.** C** mRNA expression of CXCL9, H2-D1, H2-K1 in IL11-treated (100/200 ng/ml) MC38 with/without IFNγ induction (10 ng/ml). Data presented as fold change to control group and means ± SEM from three independent experiments. *P < 0.05, **P < 0.01, *t* test. **D**, **E** Integrated graph and MFI (mean fluorescence intensity) of flow cytometry analysis on cell surface H2-Kb/Db of IL11-treated (100/200 ng/ml) MC38 with/without IFNγ induction (10 ng/ml). Data presented as fold change to control group and means ± SEM from three independent experiments. *P < 0.05, *t* test. **F** Representative images of murine intestinal organoids under IL11 stimulation. Scale bar, 400 μm.** (G)-(H)** Immunoblot of STAT1/3 (total and phosphorylated) in IL11-treated (100 ng/ml) murine intestinal organoids with/without IFNγ induction (10 ng/ml). STAT1 phosphorylation ratio was calculated. Data presented as means ± SD from three independent experiments. **P < 0.01, ***P < 0.001, *t* test. **I**–**K** mRNA expression of CXCL9, H2-D1, H2-K1 in IL11-treated (100/200 ng/ml) *Stat3*^fl/fl^ organoids (**I**), HT-29 cells (**J**) and RKO cells (**K**) with/without IFNγ induction (10 ng/ml). Data presented as fold change to control group and means ± SEM from three independent experiments. *P < 0.05, **P < 0.01, ***P < 0.001, *t* test

Previous studies have shown that IL11 functions mainly through its receptor IL11RA and GP130 to activate downstream STAT3 signaling [14]. We found that IL11 stimulation significantly increased STAT3 phosphorylation, but otherwise inhibited IFNγ-induced STAT1 phosphorylation (Fig. 3A, B). Based on ChIP-seq results conducted by Robertson et al. [33], STAT1 as transcription factor regulates expression of T cell chemokine CXCL9 and antigen presentation related molecules. In response to IL11, MC38 cells expressed lower mRNA levels of IFNγ-induced CXCL9, H2-K1 and H2-D1 (Fig. 3C). Downregulated H2-K1 and H2-D1, members of murine MHC-I molecules, were further confirmed with flow cytometry of cell surface H2-Kb/Db (Fig. 3D, E). Intestinal organoids under IL11 stimulation also presented impaired response to IFNγ and inhibited STAT1 phosphorylation (Fig. 3F–H). Lower mRNA level of IFNγ-induced CXCL9, H2-K1 and H2-D1 was detected in IL11-stimulated organoids (Fig. 3I). Similar results were found in human CRC strain HT29 and RKO in vitro (Fig. 3J, K).

### STAT3 exerts key role in IL11 inhibition of chemokine and MHC I molecule expression

**Fig. 4:**
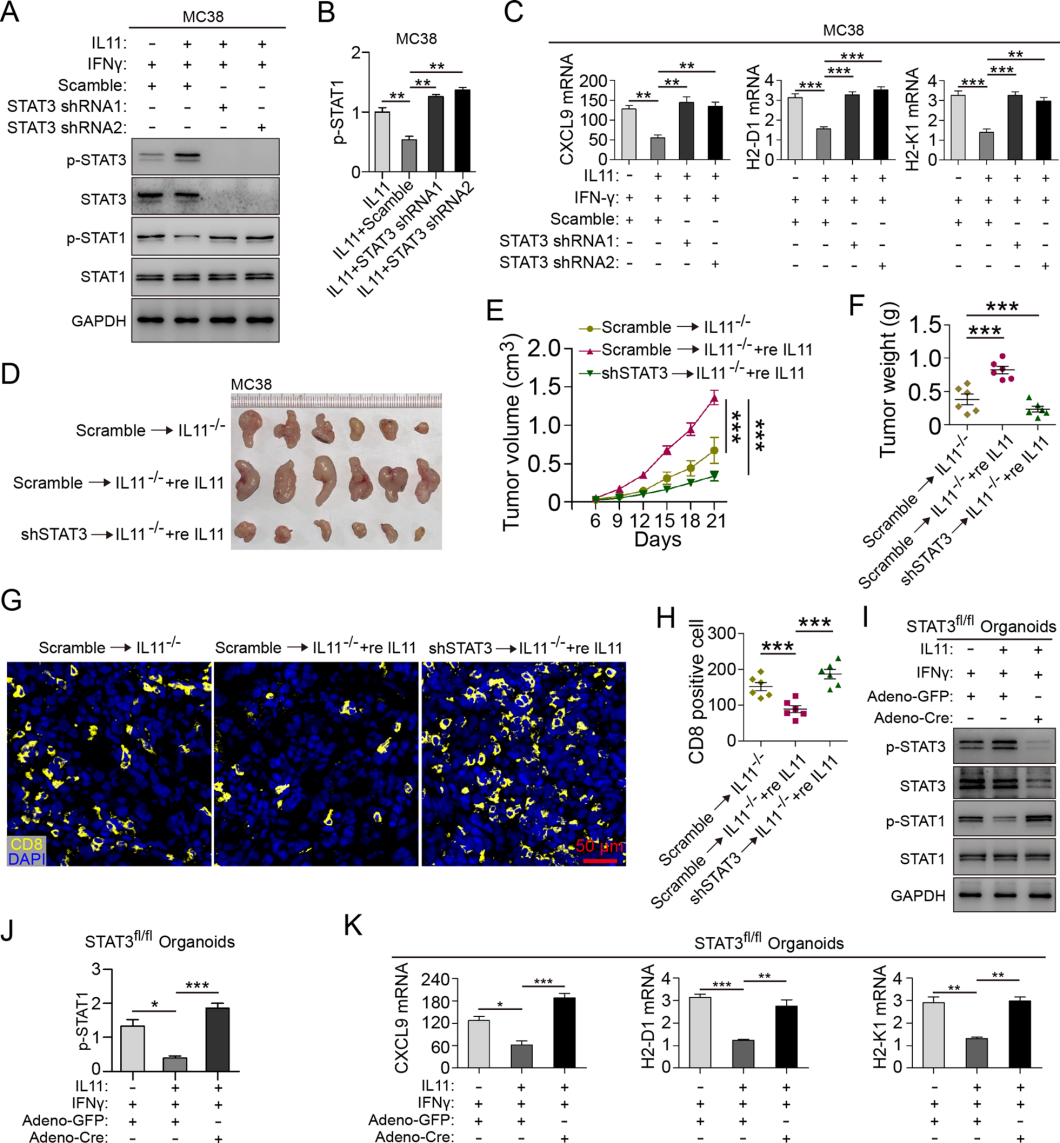
STAT3 exerts key role in IL11 inhibition of chemokine and MHC I molecule expression. **A**, **B** Immunoblot of STAT1/3 (total and phosphorylated) in IL11-treated (100 ng/ml) STAT3-knockdown MC38 with/without IFNγ induction (10 ng/ml). STAT1 phosphorylation ratio was calculated. Data presented as means ± SD from three independent experiments. *P < 0.05, **P < 0.01, ***P < 0.001, *t* test. **C** mRNA expression of CXCL9, H2-D1, H2-K1 in IL11-treated (100/200 ng/ml) STAT3-knockdown MC38 with/without IFNγ induction (10 ng/ml). Data presented as fold change to control group and means ± SEM from three independent experiments. **P < 0.01, ***P < 0.001, *t* test. **D** Representative images of STAT3-knockdown MC38 subcutaneous tumors in *Il11*^−/−^ mice with/without recombinant IL11 (reIL11, 10 mg/kg B.W.; *n* = 6 mice/group). **E** Tumor volume (cm^3^) of STAT3-knockdown MC38 subcutaneous tumors in *Il11*^−/−^ mice with/without reIL11 (10 mg/kg B.W., *n* = 6 mice/group). ***P < 0.001, two-way ANOVA. **F** Tumor weight of MC38 subcutaneous tumors in WT and *Il11*^−/−^ mice (*n* = 6 mice/group). ***P < 0.001, *t* test. **G**, **H** Immunofluorescence and cell counts of CD8 (yellow) and DAPI (blue) of STAT3-knockdown MC38 subcutaneous tumors in *Il11*^−/−^ mice with/without reIL11 (10 mg/kg B.W., *n* = 6 mice/group). Scale bar, 50 μm. ***P < 0.001, *t* test. **I**, **J** Immunoblot of STAT1/3 (total and phosphorylated) in IL11-treated (100 ng/ml) *Stat3*^fl/fl^ + Adeno-GFP/Adeno-Cre organoids with/without IFNγ induction (10 ng/ml). STAT1 phosphorylation ratio was calculated. Data presented as means ± SD from three independent experiments. *P < 0.05, **P < 0.01, ***P < 0.001, *t* test. **K** mRNA expression of CXCL9, H2-D1, H2-K1 in IL11-treated (100/200 ng/ml) *Stat3*^fl/fl^ + Adeno-GFP/Adeno-Cre organoids with/without IFNγ induction (10 ng/ml). Data presented as fold change to control group and means ± SEM from three independent experiments. *P < 0.05, **P < 0.01, ***P < 0.001, *t* test

To further clarify STAT3 in IL11 pro-tumor effect, STAT3 knockdown MC38 cells (STAT3 shRNA1/2) were constructed and treated with IL11 and IFNγ (Fig. 4A, B). In MC38-shSTAT3 cells, IL11 could not inhibit IFNγ-induced STAT1 signaling (Fig. 4A, B). STAT3 knockdown restored mRNA expression of CXCL9 and MHC-I molecules (Fig. 4C). Intraperitoneal injection of recombinant IL11 (reIL11) in tumor-bearing *Il11*^−/−^ mice boosted tumor growth, while STAT3 knockdown diminished such pro-tumor effect in vivo (Fig. 4D–F). IF analysis of CD8^+^ T cells showed that re IL11 reduced CD8^+^ T infiltration while STAT3 knockdown offset this reduction (Fig. 4G, H). Furthermore, intestinal organoids were isolated from *Stat3*^fl/fl^ conditional knockout (CKO) mice and transinfected with Adeno-Cre to establish STAT3 knockout organoids. In *Stat3*^fl/fl^ + Adeno-GFP organoids, reIL11 significantly inhibited IFNγ-induced STAT1 signaling, whereas *Stat3*^fl/fl^ + Adeno-Cre organoids showed higher IFNγ-induced STAT1 phosphorylation regardless of reIL11 treatment (Fig. 4I, J). Similarly, CXCL9, H2-D1 and H2-K1 mRNA was decreased in *Stat3*^fl/fl^ + Adeno-GFP organoids after reIL11 treatment, which could be rescued in *Stat3*^fl/fl^ + Adeno-Cre organoids (Fig. 4K). These results suggest that STAT3 exerts important role in IL11 inhibition of T-cell chemokines and MHC-I molecules.

### IL11 mutein competitively inhibit IL11 and upregulate CXCL9 and MHC-I in tumor

**Fig. 5:**
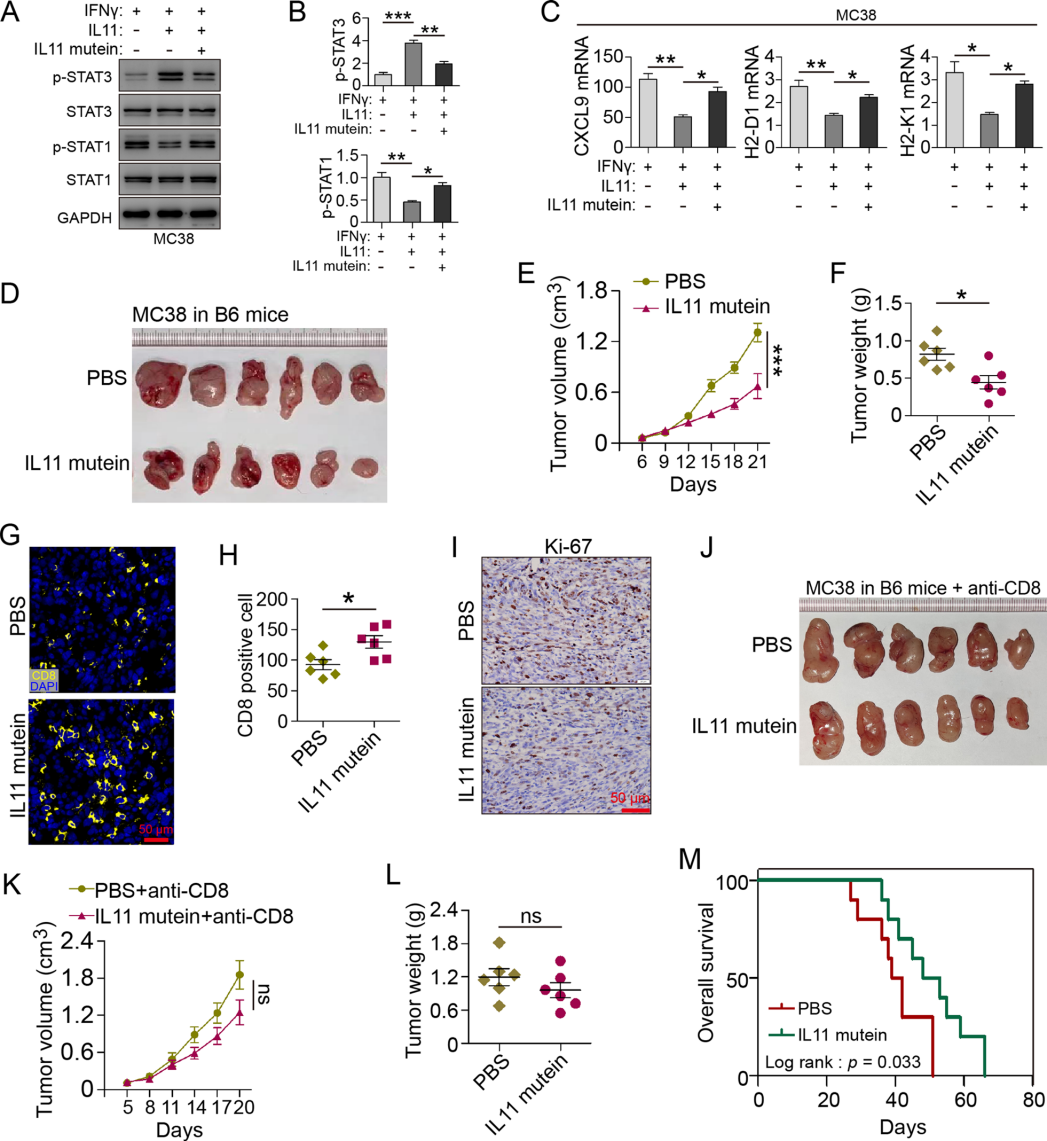
IL11 mutein competitively inhibit IL11 to upregulate CXCL9 and MHC-I in tumor. **A**, **B** Immunoblot of STAT1/3 (total and phosphorylated) in IFNγ-induced (10 ng/ml), IL11-treated (100 ng/ml) MC38 with/without IL-11 mutein (100 ng/ml). STAT1/3 phosphorylation ratio was calculated. Data presented as means ± SD from three independent experiments. *P < 0.05, **P < 0.01, ***P < 0.001, *t* test. **C** mRNA expression of CXCL9, H2-D1, H2-K1 in IFNγ/IL11-treated MC38 with/without IL11 mutein treatment (100 ng/ml). Data presented as fold change to control group and means ± SEM from three independent experiments. *P < 0.05, **P < 0.01, *t* test. **D** Representative images of MC38 subcutaneous tumors with/without IL11 mutein treatment (10 mg/kg B.W.; *n* = 6 mice/group). **E** Tumor volume (cm^3^) of MC38 tumors with/without IL11 mutein treatment (*n* = 6 mice/group). ***P < 0.001, two-way ANOVA. **F** Tumor weight of MC38 tumors with/without IL11 mutein treatment (*n* = 6 mice/group). *P < 0.05, *t* test. **G**, **H** Immunofluorescence and cell counts of CD8 (yellow) and DAPI (blue) of IL11 mutein treated MC38 tumors (*n* = 6 mice/group). Scale bar, 50 μm. *P < 0.05, *t* test. **I** Ki-67 staining of MC38 tumors with/without IL11 mutein treatment. Scale bar, 50 μm. **J** Representative images of MC38 subcutaneous tumors in CD8α-depleted WT mice with/without IL11 mutein treatment (10 mg/kg B.W.; *n* = 6 mice/group). **K** Tumor volume (cm^3^) of MC38 tumors in CD8α-depleted WT mice with/without IL11 mutein treatment (*n* = 6 mice/group). ns, no significance, two-way ANOVA. **L** Tumor weight of MC38 tumors with/without IL11 mutein treatment (*n* = 6 mice/group). ns, no significance, t-test. **M** Survival curve of MC38-i.p.-injected mice treated with/without IL11 mutein (10 mg/kg B.W.; *n* = 10 mice/group). log rank *p* = 0.033

Previous study reported PAIDY, a 5 amino acid peptide of IL11 mutein screened by Lee et al. using phage-displayed peptide library, was 20 times higher in affinity to IL11 receptor than that of wild-type counterpart, which effectively suppressed IL11/STAT3 activation [26]. IL11 mutein competitive inhibition to IL11 was validated in decreasing IL11-induced phosphorylation of STAT3 in MC38 cell line, which in turn rescued inhibited STAT1 activation to a significant extent (Fig. 5A, B). MC38 subjected to IL11 mutein showed upregulated IFNγ-induced CXCL9, H2-K1 and H2-D1, compared with wild-type IL11-treated group (Fig. 5C). Intraperitoneal injection of IL11 mutein to tumor-bearing mice also showed therapeutic effect to attenuate MC38 growth and weight in vivo (Fig. 5D–F). IL11 mutein-treated tumor showed increased CD8^+^ T cell infiltration (Fig. 5G, H) but no significant difference in Ki67^+^ cells (Fig. 5I). CD8α depletion prior to IL11 mutein treatment diminished such therapeutic effect (Fig. 5J–L). Survival analysis of MC38 intraperitoneally injected mice also indicated that IL11 mutein treatment significantly prolonged survival time in WT mice (Fig. 5M). Similar results were also conducted in CT26 models in vivo (Additional file 2: Fig. S2A–F). These results suggested that IL11 mutein competitively inhibit IL11, and IL11 mutein-treated tumor was suppressed, in which CXCL9 and MHC-I molecules were upregulated and CD8^+^ T infiltration was increased.

### IL11 is highly expressed in CRC and indicates unfavorable prognosis

**Fig. 6:**
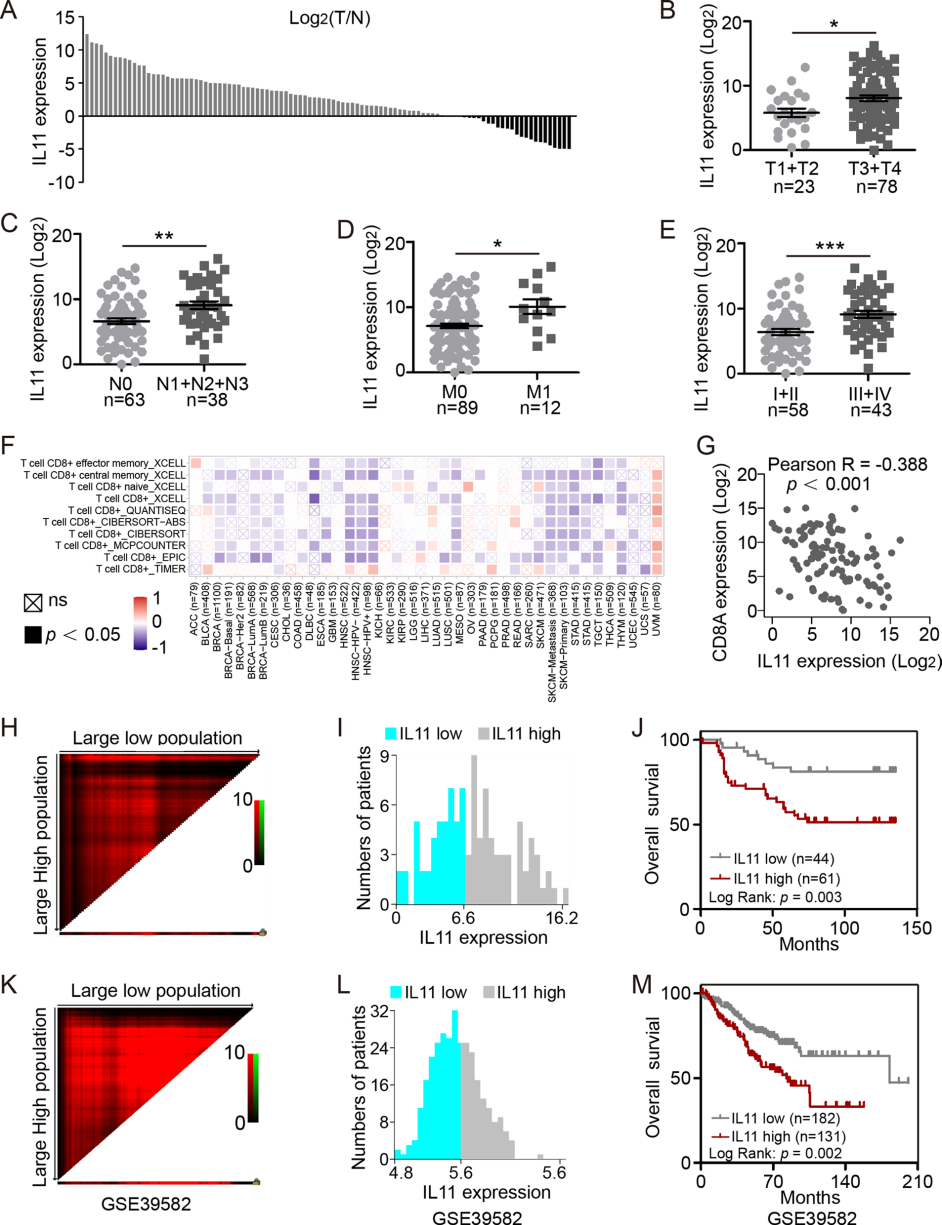
IL11 highly expressed in CRC indicates unfavorable prognosis. **A** IL11 mRNA expression in 101 paired CRC and normal samples (*n* = 101). Tumor *vs.* normal, log normalized. **B** IL11 mRNA expression in different T stages (T1 + T2 vs. T3 + T4,* n* = 23 vs. 78, AJCC ver. 8). *P < 0.05, *t* test. **C** IL11 mRNA expression in different N stages (N0 vs. N1 + N2 + N3, *n* = 63 vs. 38, AJCC ver. 8). **P < 0.01, *t* test. **D** IL11 mRNA expression in different M stages (M0 vs. M1,* n* = 89 vs. 12, AJCC ver. 8). *P < 0.05, *t* test. **(E)** IL11 mRNA expression in different AJCC stages (I + II vs. III + IV,* n* = 58 vs. 43, AJCC ver. 8). ***P < 0.001, *t* test. **F** Negative correlation between IL11 and CD8^+^ T cell infiltration in cancers (TIMER2.0). **G** IL11 and CD8A mRNA levels were detected by qRT-PCR and their correlation in our cohort (Pearson’s R = -0.388, *p* < 0.001). **H**–**J** Optimal survival cutoff of IL11 expression in local cohort using X-tile. (low vs. high, *n* = 61 vs. 44, log rank *p* = 0.003). **K**–**M** Optimal survival cutoff of IL11 expression in GSE39582 using X-tile. (low vs. high, *n* = 131 vs. 182, log rank *p* = 0.002)

101 CRC paired samples were collected from our biobank, among which 77 (76.2%) patients showed higher IL11 expression in tumor, compared with normal tissues (Fig. 6A). Analysis of clinicopathological parameters showed higher IL11 expression was detected in tumors with higher TNM stages and AJCC stages (Fig. 6B–E; Additional file 3: Table S1). TIMER2.0 website analysis indicated that IL11 was negatively correlated with CD8^+^ T cell infiltration broadly in several cancers, especially in CRC (Fig. 6F). IL11 and CD8A mRNA levels were detected by qRT-PCR and their correlation was analyzed in our cohort (Fig. 6G, Pearson’s R = − 0.388, *p* < 0.001). Using X-tile to find best cut-off to divide CRC patients into IL11-low or IL11-high group, it was demonstrated that IL11 high expression indicates poor prognosis in our cohort (Fig. 6H–J; log rank, *p* = 0.003, *n* = 44 vs*.* 61, high vs*.* low), which was also validated in dataset GSE39582 (Fig. 6K–M; log rank, *p* = 0.002, *n* = 182 vs*.* 131, high vs*.* low).

## Discussion

As a member of IL6 family, IL11 is known to promote tumor proliferation and metastasis mainly through activating STAT3 signaling [34]. IL11/STAT3 signaling effects downstream cyclin [14], which reflected in this study is that Ki67^+^ tumor cells were reduced both in IL11-deficient and IL11 mutein-treated tumors. However, after CD8^+^ T depletion, IL11 mutein-treated tumors were also suppressed, but no significant difference in Ki-67^+^ tumor cell count detected. Therefore, it is of great significance to explore the mechanism of IL11 in regulating CD8^+^ T infiltration and cytotoxicity. This study has provided evidence in clinical data that IL11 high expression indicated poor prognosis in CRC, and validated using in vitro experiments in organoids and in vivo model, to establish association between IL11 pro-tumor effect and CD8^+^ T infiltration.

This study has found that IL11 simultaneously activated STAT3 signaling and inhibited IFNγ-induced STAT1 phosphorylation and downstream T cell chemokine CXCL9 and MHC-I molecules, which lead to reduced T cell infiltration and ultimately immune invasion. CXCL9 is induced in antigen presenting cells in response to IFNγ, which amplified engraftment of tumor infiltrating lymphocytes and helped establish the ‘‘hot’’ tumor immunophenotype [35]. MHC class I molecules are the key element for surface recognition and required to anti-CTLA-4 primary response [36], and the correlation of IFNγ/STAT1/MHC-I and CD8^+^ T infiltration and cytotoxicity was well established [37–39]. Ernst et al. have revealed the balance between STAT1 and STAT3 [40], and STAT3 signaling was more stable and persistent in STAT1-null cells, which could otherwise significantly inhibit STAT1 phosphorylation, but specific mechanism has not been thoroughly elaborated. We found that IL11 could not inhibit IFNγ-induced expression of CXCL9 and MHC-I molecules in STAT3-KO cells and organoids, of which growth rate in vivo could not be accelerated by recombinant IL11 administration. These results indicated that IL11 through STAT3 inhibited the effect of IFNγ/STAT1 signaling. Ernst et al. reported that IFNγ also induced STAT3 phosphorylation [40], which was mainly conducted in IFNγ-stimulated MEF cells for 0.5 h, whereas no significant change in STAT3 phosphorylation was found during 4-h IFNγ stimulation in this study. It remains to be determined whether this is due to differences of stimulation time or cell lines.

In this study, IL11 mutein screened by Lee et al. was verified for its therapeutic effect in vivo [26], which was proved to remarkably increase CXCL9 and MHC-I molecules, thus increasing T cell infiltration tissues and antigen presentation in tumor. Putoczki et al. have also reported IL11 mutein therapeutic effect in vivo [14], but was focused on tumor Ki67 variation and lack of rescue experiment to further validate its effect on tumor proliferation. Besides Ki67 variation, our results pointed out and proved by CD8α depletion that IL11 mutein exerted therapeutic effect by increasing T cell infiltration.

Immune-suppressive role of IL11 has been revealed and discussed in recent years. Huynh et al. reported that IL11 suppressed host CD4^+^ rather than CD8^+^ T cells in the absence of canonical IL11 signaling tumor microenvironment, using CD4^+^ and CD8^+^ depletion in MC38 tumor-bearing *Il11ra*^*−/−*^ mice [25]. Compared with our findings in *Il11*^*−/−*^ mice and focus on IL11 regulation in tumor cell CXCL9 and MHC-I to increase CD8^+^ T infiltration, Huynh hinted a novel regulation besides IL11Ra for IL11 to exert immune suppression. Sumida et al. also discovered that IL11 induced differentiation of myeloid-derived suppressor cells (MDSCs) through activation of STAT3 signaling pathway [41]. These works complemented each other to illustrate a comprehensive immuno-regulatory role of IL11, which enlightened us for further investigation, because overcoming immunosuppressive tumor escape mechanisms is the key to improve immunotherapy, especially for the wide application of immune checkpoint blockade therapy in recent years.

## Conclusions

In conclusion, this study discovered a novel role of IL11 in tumor immune evasion, a finding that may provide guidance for the future exploration of IL11 targeting therapy.

## Supplementary Information

**Additional file 1: Figure S1.** Consturction and genotyping of *IL11*^−/−^ mice. **A** Schematics indicating gene editing of CRISPR/Cas9-derived knock-out allele in *Il11*^−/−^ mice. **B** Genotyping of *Il11*^−/−^ mice using DNA PCR.

**Additional file 2: Figure S2.** IL11 mutein inhibits tumor progression through promoting CD8^+^ T infiltration. **A** Representative images of CT26 subcutaneous tumors with/without IL11 mutein treatment (10 mg/kg_weight_). **B** Tumor volume (cm^3^) of CT26 tumors with/without IL11 mutein treatment. ***P < 0.001, two-way ANOVA. **C** Tumor weight of MC38 tumors with/without IL11 mutein treatment. *P < 0.05, t test. **D**, **E** Immunofluorescence and cell counts of CD8 (yellow) and DAPI (blue) of IL11 mutein treated CT26 tumors. Scale bar, 50 μm. **P < 0.01, *t* test. **F** Survival curve of CT26 intraperitoneal injection mice treated with/without IL11 mutein (10 mg/kg_weight_). log rank *p* = 0.018.

**Additional file 3: Table S1.** Clinicopathologic characteristics of CRC patients.

**Additional file 4: Table S2.** Primer sequence used for mRNA detection in human sample and cell line.

## Data Availability

The datasets supporting the conclusions of this article, GSE144735 and GSE39582 are available in Gene Expression Omnibus (GEO) database, “https://www.ncbi.nlm.nih.gov/geo/query/acc.cgi?acc=GSE144735” [32] and “https://www.ncbi.nlm.nih.gov/geo/query/acc.cgi?acc=GSE39582” [42].

## Abbreviations

CRC: Colorectal cancer
IL: Interleukin
MHC: Major histocompatibility complex
IFN: Interferon
IBD: Inflammatory bowel disease
UC: Ulcerative colitis
CAC: Colitis-associated cancer
JAK: Janus tyrosine kinases
STAT3: Signal-transducer and activated of transcription-3
CTL: Cytotoxic T lymphocyte
GEO: Gene expression omnibus
TCGA: The Cancer Genome Atlas
WT: Wild type
IHC: Immunohistochemistry
IF: Immunofluorescence
TSA: Tyramide signal amplification
HRP: Horseradish peroxidase
PBS: Phosphate-buffered saline
CKO: Conditional knockout
