# Supplementary figures and images for: Pharmacologic inhibition of IL11/STAT3 signaling increases MHC-I expression and T cell infiltration
Source: J Transl Med. 2023 Jun 26;21:416. doi: 10.1186/s12967-023-04079-6 (PMC10294373; doi:10.1186/s12967-023-04079-6)

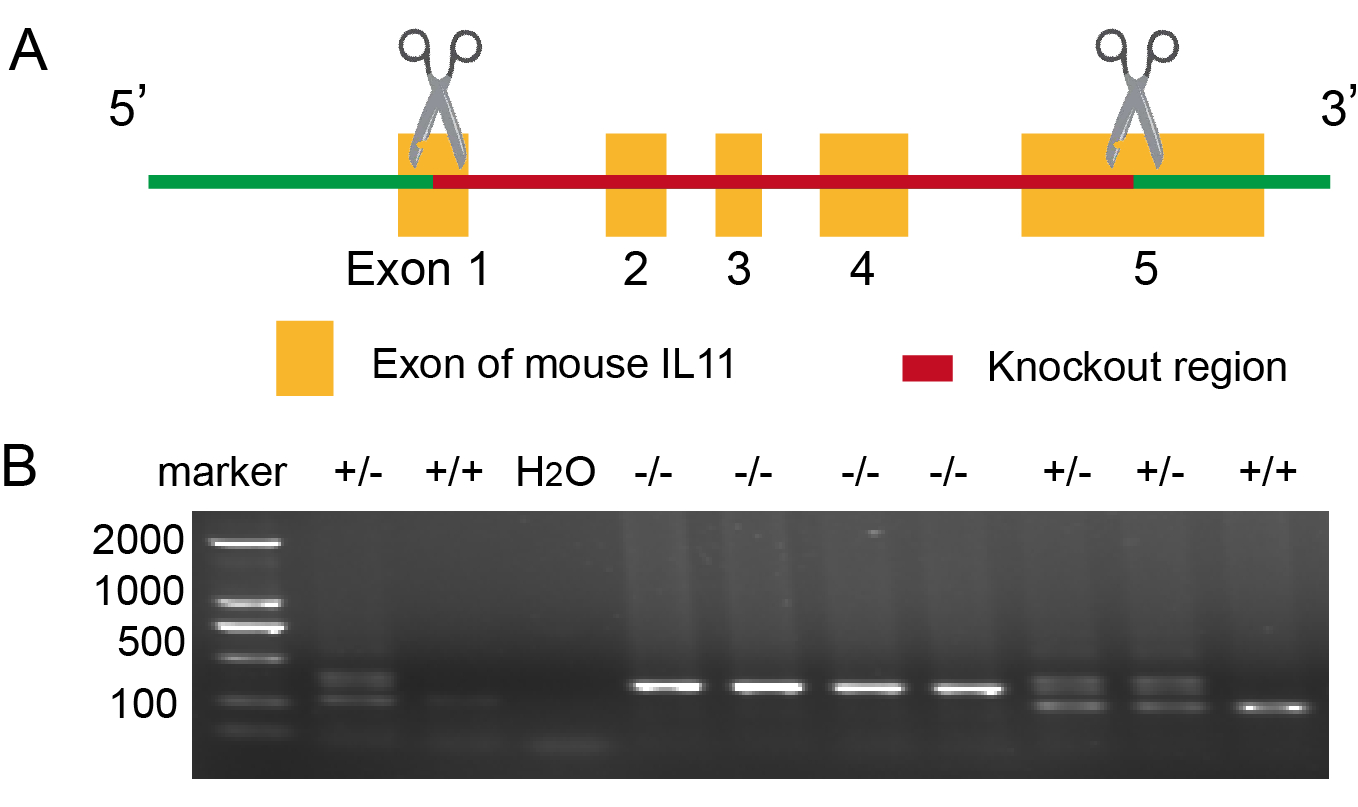

Supplement: Supplementary file 1 — Additional file 1: Figure S1. Consturction and genotyping of IL11−/− mice. A Schematics indicating gene editing of CRISPR/Cas9-derived knock-out allele in Il11−/− mice. B Genotyping of Il11−/− mice using DNA PCR. [file 12967_2023_4079_MOESM1_ESM.tif]

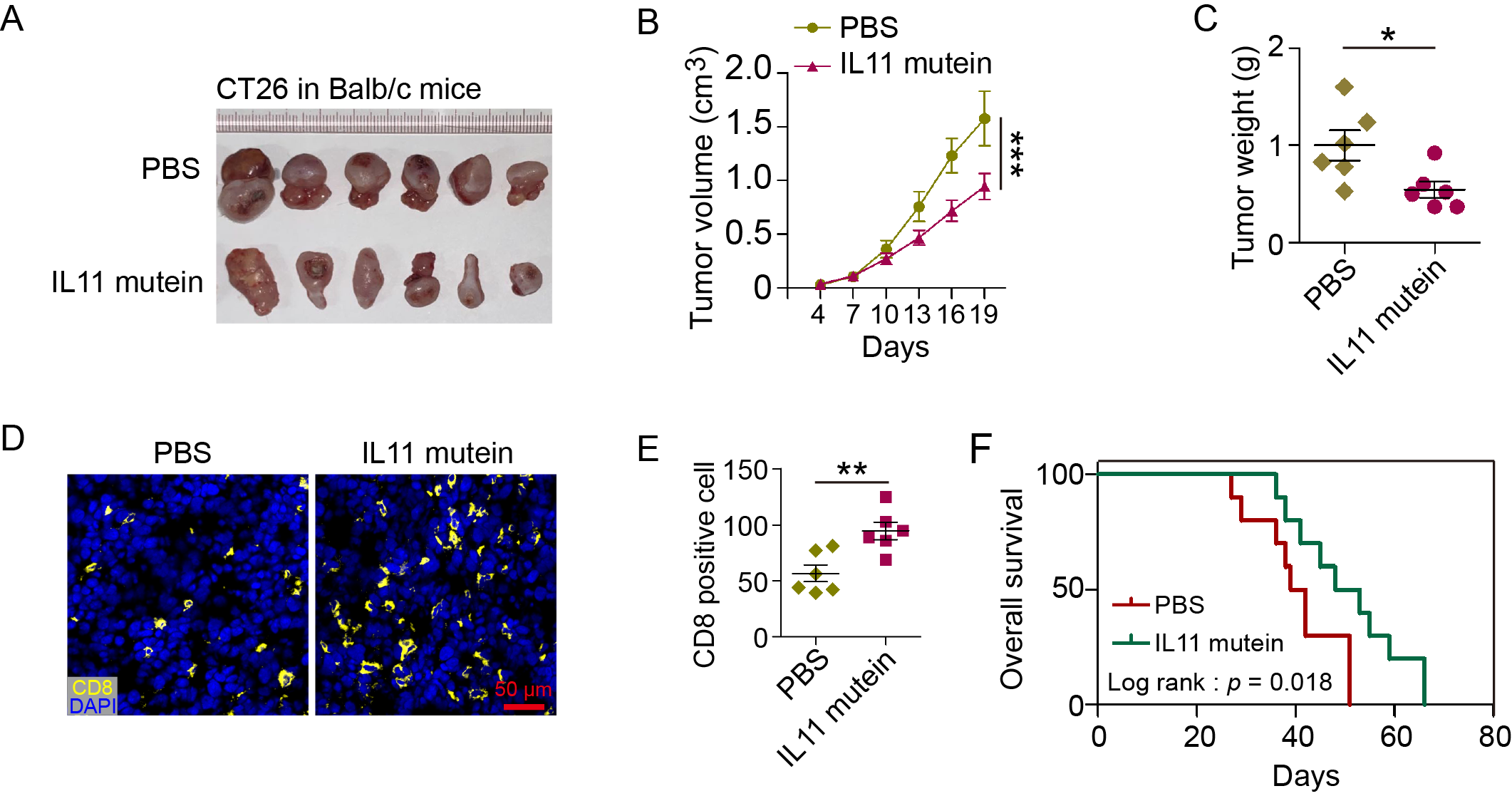

Supplement: Supplementary file 2 — Additional file 2: Figure S2. IL11 mutein inhibits tumor progression through promoting CD8+ T infiltration. A Representative images of CT26 subcutaneous tumors with/without IL11 mutein treatment (10 mg/kgweight). B Tumor volume (cm3) of CT26 tumors with/without IL11 mutein treatment. ***P < 0.001, two-way ANOVA. C Tumor weight of MC38 tumors with/without IL11 mutein treatment. *P < 0.05, t test. D, E Immunofluorescence and cell counts of CD8 (yellow) and DAPI (blue) of IL11 mutein treated CT26 tumors. Scale bar, 50 μm. **P < 0.01, t test. F Survival curve of CT26 intraperitoneal injection mice treated with/without IL11 mutein (10 mg/kgweight). log rank p = 0.018. [file 12967_2023_4079_MOESM2_ESM.tif]
